# Antenatal placental assessment in the prediction of adverse pregnancy outcome after reduced fetal movement

**DOI:** 10.1371/journal.pone.0206533

**Published:** 2018-11-05

**Authors:** Lucy E. Higgins, Jenny E. Myers, Colin P. Sibley, Edward D. Johnstone, Alexander E. P. Heazell

**Affiliations:** 1 Maternal and Fetal Health Research Centre, Faculty of Biology, Medicine and Health, University of Manchester, Manchester, United Kingdom; 2 St. Mary's Hospital, Manchester University NHS Foundation Trust, Manchester Academic Health Science Centre, Manchester, United Kingdom; University of Liverpool, UNITED KINGDOM

## Abstract

**Objective:**

To assess the value of *in utero* placental assessment in predicting adverse pregnancy outcome after reported reduced fetal movements (RFM).

**Method:**

A non-interventional prospective cohort study of women (N = 300) with subjective RFM at ≥28 weeks’ gestation in singleton non-anomalous pregnancies at a UK tertiary maternity hospital. Clinical, sonographic (fetal weight, placental size and maternal, fetal and placental arterial Doppler) and biochemical (maternal serum hCG, hPL, progesterone, PlGF and sFlt-1) assessment was conducted. Multiple logistic regression identified combinations of measurements (models) most predictive of adverse pregnancy outcome (perinatal mortality, birth weight <10^th^ centile, five minute Apgar score <7, umbilical arterial pH <7.1 or base excess <-10, neonatal intensive care admission). Models were compared by test performance characteristics (ROC curve, sensitivity, specificity, positive/negative predictive value, positive/negative likelihood ratios) against baseline care (estimated fetal weight centile, amniotic fluid index and gestation at presentation).

**Results:**

61 (20.6%) pregnancies ended in adverse outcome. Models incorporating PlGF/sFlt-1 ratio and umbilical artery free loop Doppler impedance demonstrated modest improvement in ROC area for adverse outcome (baseline care 0.69 vs. proposed models 0.73–0.76, p<0.05). However, there was little improvement in other test characteristics (baseline vs. best proposed model: sensitivity 21.7% [95% confidence interval 13.1–33.6] vs. 35.8%% [24.4–49.3], specificity 96.6% [93.4–98.3] vs. 94.7% [90.7–97.0], PPV 61.9% [40.9–79.3] vs. 63.3% [45.5–78.1], NPV 82.8% [77.9–86.8] vs. 85.2% [80.0–89.2], positive LR 6.3 [2.8–14.6] vs. 6.7 [3.4–3.3], negative LR 0.81 [0.71–0.93] vs. 0.68 [0.55–0.83]) and wide confidence intervals. Negative post-test probability remained high (16.7% vs. 14.0%).

**Conclusion:**

Antenatal placental assessment may improve identification of RFM pregnancies at highest risk of adverse pregnancy outcome but further work is required to understand and refine currently available outcome definitions and diagnostic techniques to improve clinical utility.

## Introduction

Up to one in 250 pregnancies in high-income countries ends in stillbirth [1], one third of which occur ≥37 weeks’ gestation [2–5] and are potentially preventable by delivery without incurring significant neonatal complications. Women who present with reduced fetal movements (RFM) are an “at risk” population, with increased risk of stillbirth and fetal growth restriction (FGR) [6–8].

Currently there is no accurate predictive clinical test identifying which pregnancies are at highest risk of fetal death [9], leading to varied practice [10–12]. Standard care, as defined by the Royal College of Obstetricians and Gynaecologists, is cardiotocograph, and assessment of fetal size and liquor volume; umbilical artery Doppler assessment is not currently recommended [13]. No further guidance is given regarding ongoing surveillance of these pregnancies provided fetal movements return to normal. Where repeated episodes occur, particularly approaching and beyond term gestation, delivery is often expedited [14]. Yet in the absence of intervention, the interval between presentation with RFM and delivery may be several weeks long. Babies of an apparently appropriate size at initial presentation with RFM may subsequently experience impaired intrauterine growth trajectory, or fetal compromise during the physiological stress of labour. These can be clinical features of placental insufficiency.

Ex vivo placentas from RFM pregnancies with adverse pregnancy outcome display structural and functional features of placental insufficiency similar to those of stillborn infants or live born FGR infants [15–17]. Relevant aspects of placental structure and function can be assessed by ultrasound (e.g. placental diameter and volume [18], tissue vascularity [19]) or by maternal circulation concentration of placentally derived hormones (e.g. human placental lactogen (hPL) and human chorionic gonadotrophin (hCG)). Therefore, placental assessment is proposed as a means to improve prediction of adverse pregnancy outcome, by detection of placental insufficiency [20].

We hypothesised that antenatal placental assessment would improve the prediction of RFM pregnancies at highest risk of placentally-derived adverse pregnancy outcome compared with baseline care. We aimed to test the diagnostic accuracy of various models of predicting adverse pregnancy outcome following RFM.

## Materials and methods

A prospective longitudinal cohort study of women attending the antenatal service with a reduction in fetal movements was performed in accordance with the Declaration of Helsinki 1975 (revised 2013), following ethical approval from Greater Manchester North West Research Ethics Committee (11/NW/0650).

### Participant recruitment

Women with singleton pregnancies of ≥28 weeks’ gestation presenting with a subjective reduction in perceived fetal activity [13] between January 2012 and May 2014 were prospectively approached during the process of routine clinical evaluation (completed within a maximum 72h from presentation) until 300 women provided written informed consent. Exclusion criteria were; immediate fetal compromise on cardiotocograph, fetal abnormality or pre-existing hypertension or diabetes. Patient records were contemporaneously accessed with patient consent to record the required background data.

### Fetoplacental assessment *in utero*

Fetal wellbeing was assessed by a single individual (LH) according to unit policy / Royal College of Obstetricians and Gynaecologists guidelines [13] as follows: estimated fetal weight (EFW) centile (Bulk centile calculator v6.7 (UK), Gestation Network, Birmingham, UK), four-quadrant amniotic fluid index [21] and quantification of vascular impedance at the middle third of the umbilical artery (UAD-Free) by pulsatility index (PI) and resistance index (RI) [22]. These results were revealed to the clinical team. A maximum of 45 minutes scanning time (shorter if patient discomfort occurred) was permitted, with measurements required for routine care being prioritised above research measurements.

Further ultrasound measurements were made as follows: curvilinear placental length, width, maximal depth and volume (VOCAL technique with 30° rotation angle, 4Dview software v.5 (GE Healthcare)) [18], vascular impedance at the umbilical artery abdominal (UAD-Abdomen) and placental (UAD-Placenta) insertion points [23, 24], mean impedance of four chorionic plate arteries and intraplacental arteries respectively, transabdominal uterine artery Doppler PI, RI and notch status [25], middle cerebral artery PI, RI and peak systolic velocity [26–30]. Maternal venous blood (BD Vacutainer, Franklin Lakes, US) was processed to obtain serum and stored at -80°C. Serum concentrations of hCG*, hPL*, progesterone*, placental growth factor^#^ (PlGF) and soluble fms-Like tyrosine kinase-1^#^ (sFlt-1) were measured using enzyme-linked immunosorbant assay kits in accordance with the manufacturer’s instructions (*DRG International, Springfield, USA. ^#^R&D systems, Abingdon, UK).

### Outcome definition and data collection

No relevant core outcome set was identified for outcome reporting. Adverse pregnancy outcome was defined as a composite of any of the following: stillbirth or neonatal death, individualised birth weight centile <10 (Bulk centile calculator v.6.7 (UK) (Gestation Network, Birmingham, UK), five minute Apgar <7, umbilical artery pH<7.1 or base excess<-10 or admission to neonatal intensive care within 24 hours of birth in accordance with previous studies [31, 32]. Normal outcome was defined as the absence of these adverse outcomes and does not necessarily indicate that other non-placentally derived adverse outcomes were not present.

Following assessment by the research team, participants returned to routine care, unless an abnormality in baseline care measurements was identified, in which case this was reported to the clinical team providing care for women. Importantly for this observational study, the research team were not involved in determining subsequent antenatal or intrapartum care. The research team were notified of the patient’s delivery, and reviewed the patient’s case notes following discharge from hospital to collect outcome data or 28 days after expected date of delivery if no notification had been received. If there was no record of delivery at the hospital delivery details were sought from the patient’s General Practitioner. If no outcome details could be obtained from this source, the participant was deemed “lost to follow up”.

### Statistical analysis

Anonymised data pertaining to the study participants, with the exclusion of potential patient identifiers [33], is available online [34]. Statistical analysis was performed using Stata 13 (StataCorp, College Station, USA). Data of participants and non-participants were compared by univariate analysis (Student’s t test, Mann-Whitney U test and Chi squared test with Yates’ correction as required for parametric, non-parametric and categorical data respectively). Where data were missing, the denominator was reduced accordingly. Sonographic accuracy of fetal weight estimates within seven days of delivery was assessed by Bland Altman plot (bias, 95% confidence intervals). Rate of decline (centile difference/scan to delivery interval) for the whole cohort was presented as median (interquartile range) and compared between those with and without adverse outcome by Mann-Whitney U test.

Participant demographics, past medical and obstetric histories, RFM episode features, sonographic and endocrine variables were analysed, singularly or in combination, by pregnancy outcome. Variables were rejected where univariate analysis demonstrated lack of potential association with adverse pregnancy outcome (*a priori* threshold p≥0.10). Next, adverse pregnancy outcome odds ratios for the remaining predictors (transformed where non-parametric) were calculated following adjustment for association with elements of current standard care (EFW centile, amniotic fluid index) and for gestational age (as clinical care tends to vary with gestational age [14], and to mitigate gestational change in the examined predictors). These combined three continuous variables (gestational age, EFW centile, amniotic fluid index) are here after referred to as the baseline model. Variables with statistically significant adjusted odds ratios for adverse pregnancy outcome (p<0.05) were rationalised by factor analysis to ensure only independent predictors from each category of variables were included in the model development. Only complete datasets were used in the regression analyses. Remaining predictors were combined in multiple logistic regression to identify combinations of variables (predictive models) that demonstrated superior receiver operating characteristic (ROC) curve area than baseline care (p<0.05). Proposed models were rejected if a) missing data reduced the number of adverse pregnancy outcome events in the model to below 10/variable, or b) if the area under the ROC curve was not significantly different to the baseline model.

The proposed models were compared against baseline care by test characteristics (sensitivity, positive and negative predictive values, positive and negative likelihood ratios and post-test probabilities) aiming to achieve a positive likelihood ratio>10 and negative likelihood ratio<0.2. Test characteristics were presented alongside 95% confidence intervals. The study is reported according to Standards for Reporting of Diagnostic accuracy studies (STARD) guidelines (S1 Table). Given the poor discrimination of angiogenic markers >37 weeks in previous studies [35, 36], a sensitivity analysis was performed to assess the model performance in women who presented at >37 weeks.

Based on an expected adverse pregnancy outcome rate of 20% demonstrated in previous cohorts of RFM pregnancies [31], with 5% loss to follow up, N = 300 participants was anticipated to provide sufficient power to adjust individual adverse pregnancy outcome risk using up to six predictive risk factors/measurements.

## Results

**Fig 1:**
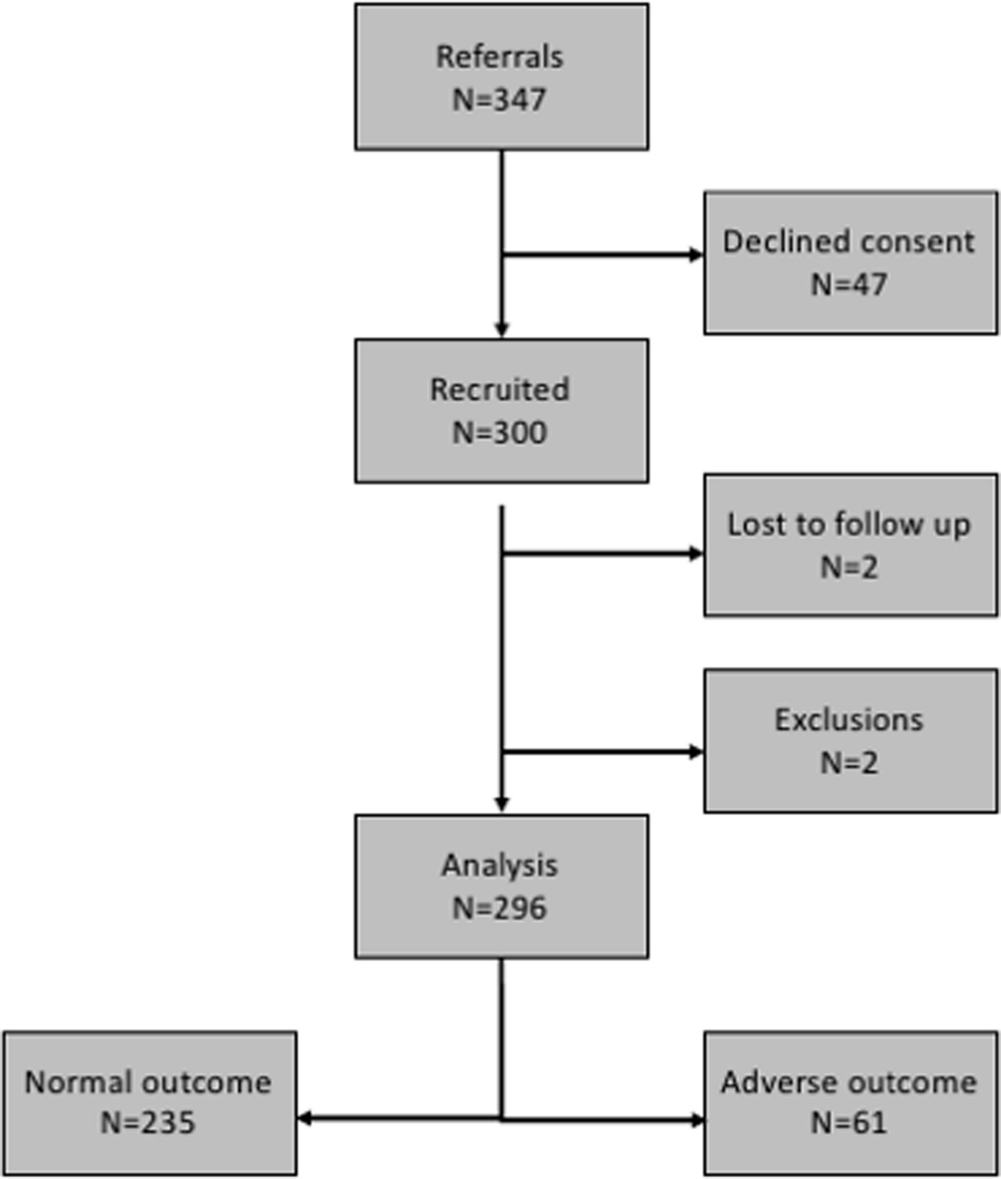
Flow of participants through the FEMINA2 study.

**Fig 2:**
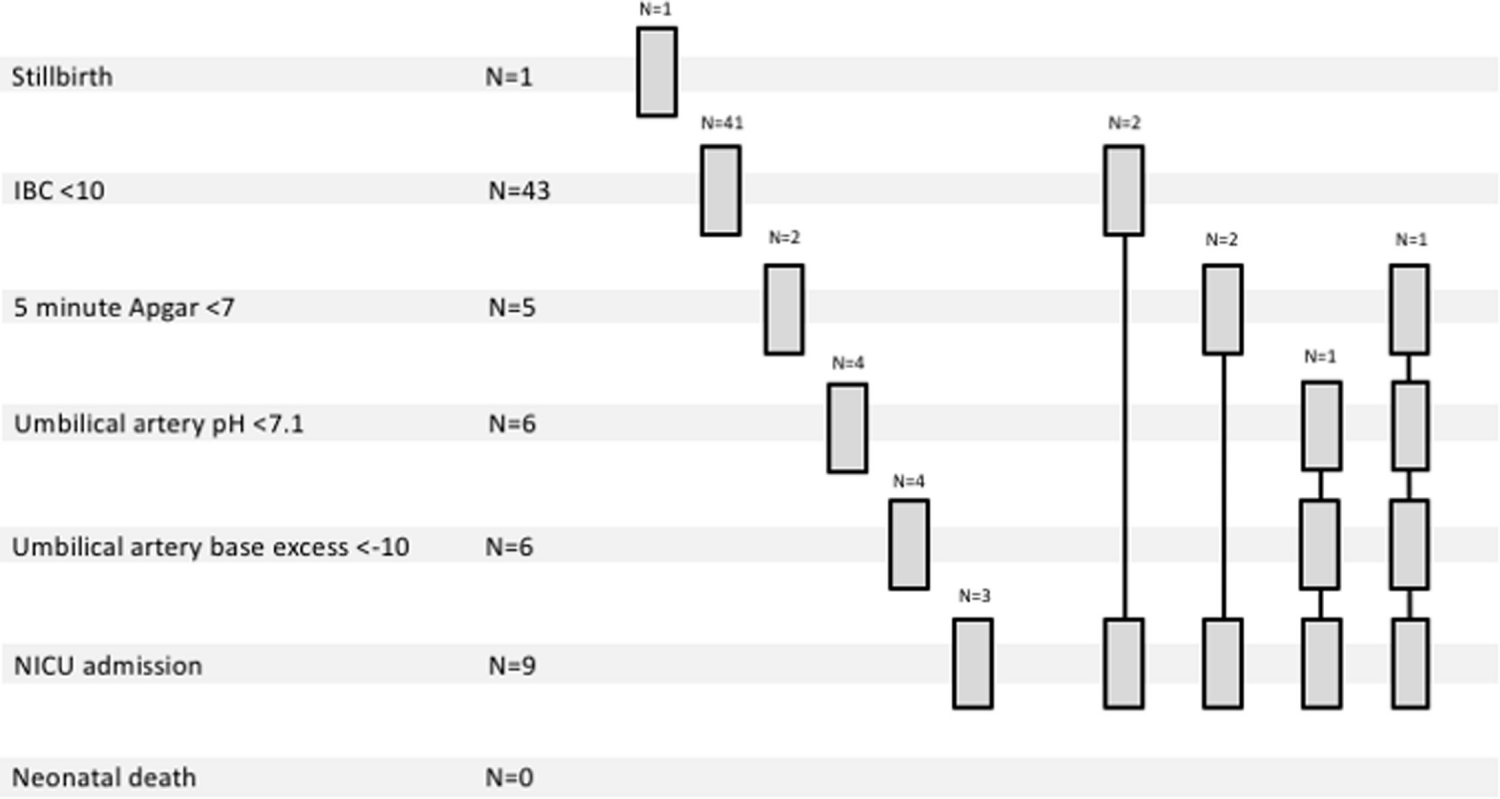
Breakdown of adverse pregnancy outcomes within the FEMINA2 study cohort. Adverse pregnancy outcome was diagnosed on the basis of the occurrence of one or more classifier of adverse outcome: stillbirth, individualised birth weight centile (IBC)<10, five minute Apgar score<7, umbilical arterial pH<7.1 or base excess<-10, admission to neonatal intensive care unit (excluding for fetal abnormality, jaundice or sepsis) or neonatal death before discharge.

347 women were approached. Compared with non-participants (N = 47), participants (N = 300) were more likely to be White European (68.0% vs. 36.2%, p<0.001) and of lower parity (median 0 (interquartile range 0–1) vs. 0 (0–2), p = 0.039). Four participants were excluded (N = 2 lost to follow up, N = 2 postnatal diagnosis of fetal abnormality). Fig 1 shows the flow of participants through the study. Of 296 pregnancies analysed, 61 (20.6%) resulted in adverse pregnancy outcome: (non-exclusive categories: stillbirth N = 1, birth weight centile <10 N = 43, five minute Apgar <7 N = 6, umbilical artery pH <7.1 N = 6, umbilical artery base excess <-10 N = 6, neonatal intensive care unit admission N = 9, neonatal death N = 0) (Fig 2). Following admission to the neonatal intensive care unit (admissions primarily for sepsis, jaundice and fetal abnormality were not counted) babies spent a median of 6 (6–7) days in the neonatal unit.

Those experiencing adverse pregnancy outcome were more likely to report a longer duration of absent movements (p = 0.014), develop pregnancy-induced non-proteinuric hypertension (p<0.001), deliver prior to 34 weeks’ gestation (p = 0.047), or to be delivered for presumed fetal distress (p = 0.004) (Table 1) than those without adverse outcome (235/296 [79.4%]). Trends were also shown in a tendency for those experiencing adverse pregnancy outcome to report a longer total duration of RFM (p = 0.073), deliver prior to 37 weeks’ gestation (overall p = 0.071; iatrogenic p = 0.060), deliver by pre-labour caesarean section (p = 0.092), to experience static growth above the 10^th^ customised centile (p = 0.067) and to be clinically diagnosed with placental abruption (2/61 [3.3%], p = 0.050).

**Table 1:** Comparison of women participating in the FEMINA2 trial and their pregnancy outcomes.

Pregnancy outcome N	Normal 235	Adverse 61	p
**Maternal characteristics**			
**Age** (Years)	29 (26–33)	30 (25–33)	0.60
**Ethnicity**			0.91
White European Asian Black Other	152/235 (65%) 39/235 (17%) 31/235 (13%) 13/235 (5.5%)	41/61 (67%) 11/61 (18%) 6/61 (9.8%) 3/61 (4.9%)	
**BMI** (kg/m^2^)	25.4 (22.7–29.1)	26.8 (23.0–31.3)	0.20
Parity (Number)	0 (0–1)	0 (0–1)	0.69
**Reduced fetal movement episode characteristics**			
**Episode number** (Number)	1 (1–1)	1 (1–1)	0.72
**Episode duration** (Hours)	36 (18–72)	48 (24–72)	0.073
**Absent movements (any)** If absent, hours	59/209 (28%) 10 (4–12)	13/56 (23%) 16 (12–24)	0.45 0.014
**Gestation at presentation** (Weeks^+days^)	37^+2^ (33^+5^–39^+2^)	36^+5^ (31^+4^–38^+6^)	0.21
**Resolved**	95/216 (44%)	21/56 (38%)	0.38
**CTG:**			
**Baseline rate (bpm)** **Variability <5bpm** **Accelerations** **Decelerations** **Movements/min**	137 ± 8 0/204 (0.%) 192/204 (94%) 13/204 0.28 (0.12–0.69)	136 ± 8 0/53 (0%) 48/53 (91% 6/53 0.31 (0.11–0.57)	0.44 1.00 0.35 0.22 0.47
**Pregnancy complications**			
**Further RFM** No. further episodes	52/235 (22%) 1 (1–2)	18/61 (30%) 1 (1–2)	0.23 0.64
**Preeclampsia**	7/234 (3.0%)	3/61 (4.9%)	0.46
**Pregnancy induced hypertension**	1/234 (0.4%)	4/61 (6.6%)	0.00095
**Gestational proteinuria**	2/234 (0.9%)	1/61 (1.6%)	0.59
**Static growth** Of which EFW>10^th^ centile	8/234 (3.4%) 7/8 (88%)	4/61 (6.6%) 3/4 (75%)	0.27 0.067
**Oligohydramnios**	3/234 (1.3%)	1/61 (1.6%)	0.83
**Polyhydramnios**	8/234 (3.4%)	1/61 (1.6%)	0.47
**Gestational diabetes**	4/234 (1.7%)	1/61 (1.6%)	0.97
**Large for gestational age**	7/234 (3.0%)	0/61 (0.0%)	0.17
**Obstetric cholestasis**	5/234 (2.1%)	0/61 (0.0%)	0.25
**Antepartum haemorrhage**	6/234 (2.6%)	1/61 (1.6%)	0.67
**Abruption**	0/234 (0.0%)	2/61 (3.3%)	0.050
**Chorioamnionitis**	4/234 (1.7%)	0/61 (0.0%)	0.30
Delivery characteristics*			
**Delivery interval** (Days)	15 (5–39)	14 (6–46)	0.99
**Gestation at delivery** (Weeks^+days^)	40^+1^ (38+6–41^+1^)	40^+0^ (39+0–40^+5^)	0.19
**Preterm delivery:**			
<34 weeks Of which spontaneous <37 weeks Of which spontaneous	1/235 (0.4%) 0/1 (0.0%) 12/235 (5.1%) 7/12 (58%)	2/61 (3.3%) 0/2 (0.0%) 7/61 (11%) 1/7 (14%)	0.047 1.00 0.071 0.060
**Induction of labour**	104/235 (44%)	28/61 (46%)	0.82
**Laboured**	208/235 (89%)	49/61 (80%)	0.092
**Caesarean Section** Pre-labour In labour	44/235 (19%) 27/235 (12%) 17/235 (7%)	15/61 (25%) 8/61 (13%) 7/61 (11%)	0.32 0.73 0.28
**Delivery for fetal distress**	33/235 (14%)	18/61 (30%)	0.0044
Outcome Characteristics*			
**Male infant**	128/235 (54%)	29/61 (48%)	0.33
**Birth weight centile** Centile difference Centile difference/day	47.6 (27.5–71.5) -11.5 (-27.2–2.0) -0.4 (-2.3–0.08)	8 (3.7–24.0) -21.0 (-43.5–-6.5) -0.9 (-2.9–-0.3)	<0.0001 0.0004 0.014
**5 min Apgar score**	10 (10–10)	10 (9–10)	0.0004
**Umbilical Arterial pH**	7.24 (7.20–7.29)	7.20 (7.13–7.25)	0.0020
**Umbilical Artery Base Excess**	-4.3 (-6.9–-2.1)	-5.9 (-8.8–-3.0)	0.033

Continuous data are expressed as median (interquartile range) and compared by Mann-Whitney U Test. Categorical data are expressed as number (%) and compared by Chi Squared test (with Yates’ correction as required). Statistical significance was set at the level of p<0.05. CTG = cardiotocograph. BPM = beats per minute. RFM = reduced fetal movements. EFW = estimated fetal weight. Birth weight centile = individualised birth weight centile (Bulk centile calculator v6.7 (UK) (Gestation Network, Birmingham, UK).

*Two participants and one non-participants were lost to follow up, a further two participants were excluded from analysis of pregnancy outcome following postnatal diagnosis of fetal abnormality. Where clinical data is missing the denominator is accordingly reduced.

**Fig 3:**
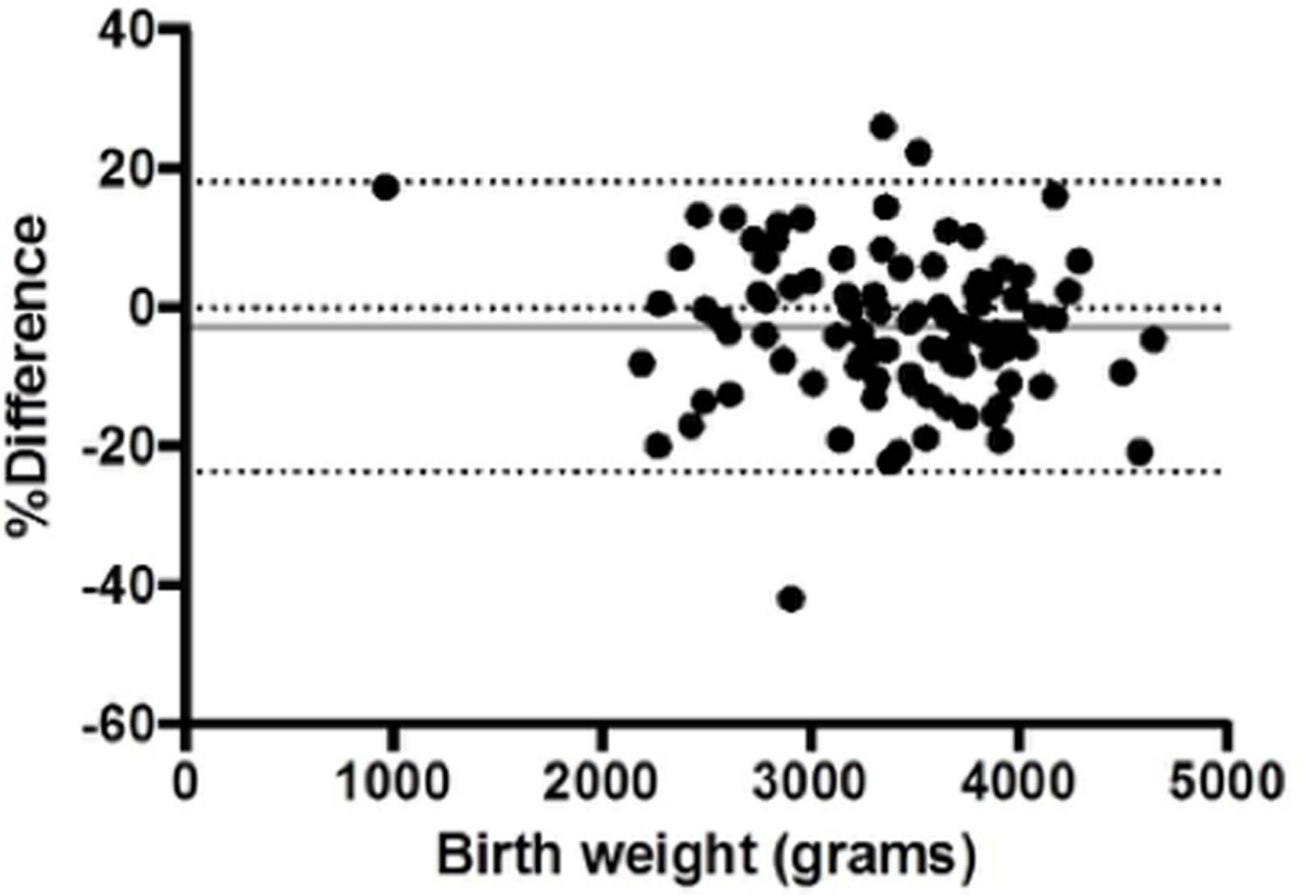
Accuracy of fetal weight estimation within seven days of birth. Bland-Altman plot comparing the difference between estimated fetal weight and actual birth weight (y axis) to the birth weight itself (x axis) for deliveries occurring between 0–7 days from study enrolment. This shows minimal systematic error in estimated fetal weight (grey solid line). The dotted lines show the limits of agreement.

The accuracy of EFW estimation was good with a mean bias of -2.8% (Fig 3). However, within the whole cohort, a median of 13.1 centiles/baby were dropped between scan and delivery. Adverse outcome pregnancies demonstrated greater overall centile decline (p<0.001) despite no significant difference in scan to delivery interval (p = 0.99), indicating a steeper decline in centiles when adjusted for scan to delivery interval (p = 0.014).

Following our predetermined analysis strategy, from 107 variables, 30 demonstrated potential univariate association with adverse pregnancy outcome (p<0.10; S2 Table). After adjustment for EFW centile, amniotic fluid index and gestation (elements of “baseline care”), nine variables were considered independently associated with adverse pregnancy outcome (p<0.05; Table 2). These included three variables relating to PlGF (total PlGF, PlGF/sFlt-1 ratio and “free-PlGF”), five measures of impedance to flow along the umbilical artery (UAD-abdomen RI, UAD-Free PI and RI, and the UAD-placenta/UAD-Free ratios for PI and RI) and one variable relating to brachiocephalic blood diversion (Middle Cerebral Artery Doppler/UAD-Free RI ratio). Following factor analysis quantifying covariance of linked variable, the PlGF/sFlt-1 ratio, UAD-Free PI and RI and Middle Cerebral Artery Doppler/UAD-Free RI ratio were preferentially retained above related variables. Doppler measures (UAD-Free PI, RI and Middle cerebral artery Doppler/UAD-Free RI ratio) were introduced into the models individually, and were not used in combination.

**Table 2:** Odds ratios for adverse pregnancy outcome by individual differentially distributed variables.

Variable	Inc	OR	95% CI	p	aOR	95% CI	p
**Baseline Model**							
Gestation (week)	1	0.96	(0.89–1.04)	0.32	0.91	(0.83–1.00)	0.033
Post-mature presentation	Binary	0.47	(0.19–1.17)	0.11	0.61	(0.21–1.78)	0.34
**Estimated fetal weight centile**	**5**	**0.89**	**(0.84–0.93)**	**<0.0001**	**0.89**	**(0.84–0.94)**	**<0.0001**
Amniotic Fluid Index (cm)	1	0.93	(0.86–1.00)	0.090	0.90	(0.82–0.99)	0.051
**Maternal characteristics**							
Height (cm)	1	0.021	(0.002–1.74)	0.087	0.032	(0.00026–3.86)	0.16
Miscarriages (number)	1	1.17	(0.93–1.46)	0.11	1.18	(0.93–1.50)	0.12
Significant Past Medical History	Binary	2.37	(0.99–5.66)	0.052	1.92	(0.75–4.91)	0.17
Previous birth<10^th^ centile	Binary	2.23	(0.98–5.08)	0.057	1.42	(0.59–3.45)	0.47
**RFM Characteristics**							
Duration (days)	1	1.01	(0.95–1.09)	0.056	0.98	(0.91–1.06)	0.35
**Placental Size Assessment**							
Length (cm)	1	0.89	(0.80–0.99)	0.036	0.93	(0.84–1.04)	0.22
Width (cm)	1	0.91	(0.82–1.01)	0.082	0.97	(0.87–1.09)	0.64
**Placental Vascular Assessment**							
UAD-Abdomen PI	0.1	1.21	(1.04–1.41)	0.009	1.15	(0.98–1.36)	0.063
**UAD-Abdomen RI**	**0.1**	**2.16**	**(1.37–3.41)**	**0.001**	**1.95**	**(1.17–3.27)**	**0.013**
**UAD-Free PI**	**0.1**	**1.26**	**(1.09–1.45)**	**0.001**	**1.26**	**(1.06–1.49)**	**0.013**
UAD-Free PI>95^th^ centile	Binary	3.14	(1.41–7.00)	0.005	2.39	(0.98–5.81)	0.54
**UAD-Free RI**	**0.1**	**1.92**	**(1.32–2.78)**	**0.001**	**2.05**	**(1.27–3.29)**	**0.005**
**UAD-Free RI>95**^**th**^ **centile**	**Binary**	**12.71**	**(2.50–64.67)**	**0.002**	**6.01**	**(1.02–35.49)**	**0.048**
Chorionic plate artery RI	0.1	1.61	(1.01–2.56)	0.045	1.36	(0.75–2.47)	0.32
Intraplacental artery PI	0.1	1.34	(1.05–1.70)	0.016	1.26	(0.97–1.65)	0.09
Intraplacental artery RI	0.1	1.68	(1.01–2.78)	0.044	1.46	(0.81–2.62)	0.22
Chorionic plate artery: UAD-Abdomen PI ratio	0.1	0.82	(0.67–1.01)	0.08	0.82	(0.65–1.03)	0.11
**UAD-Placenta: UAD-Free PI ratio**	**0.1**	**0.86**	**(0.72–1.02)**	**0.050**	**0.82**	**(0.67–0.99)**	**0.026**
**UAD-Placenta: UAD-Free RI ratio**	**0.1**	**0.77**	**(0.59–1.00)**	**0.039**	**0.72**	**(0.54–0.97)**	**0.028**
**Chorionic plate artery: UAD-Free PI ratio**	**0.1**	**0.82**	**(0.68–0.98)**	**0.029**	**0.79**	**(0.64–0.97)**	**0.026**
**Chorionic plate artery: UAD-Free RI ratio**	0.1	0.77	(0.58–1.02)	0.057	0.73	(0.54–1.00)	0.052
**Placental Endocrine Assessment**							
Log[hPL]	1	0.54	(0.15–1.98)	0.085	0.77	(0.15–3.80)	0.56
**Log[PlGF] (pg/L)**	**1**	**0.64**	**(0.42–0.97)**	**0.033**	**0.58**	**(0.36–0.92)**	**0.021**
**Log[PlGF: sFlt-1]**	**1**	**0.81**	**(0.65–1.00)**	**0.050**	**0.64**	**(0.47–0.85)**	**0.003**
**Log[Free PlGF] (pg/L)**	**1**	**0.85**	**(0.74–0.99)**	**0.037**	**0.76**	**(0.63–0.92)**	**0.004**
**Brachiocephalic Blood Diversion**							
MCA: UAD-Abdomen PI ratio	0.1	0.91	(0.82–1.00)	0.066	0.191	(0.82–1.02)	0.16
MCA: UAD-Abdomen RI ratio	0.1	0.71	(0.55–0.90)	0.005	0.75	(0.58–1.00)	0.052
MCA: UAD-Free PI ratio	0.1	0.94	(0.88–1.01)	0.059	0.94	(0.88–1.01)	0.12
**MCA: UAD-Free RI ratio**	**0.1**	**0.77**	**(0.64–0.94)**	**0.0060**	**0.80**	**(0.66–0.98)**	**0.041**
MCA: Intraplacental artery RI	0.1	0.85	(0.71–1.02)	0.085	0.89	(0.73–2.70)	0.32

Unadjusted (OR) and adjusted (aOR) odds ratios are presented with 95% confidence intervals (95% CI) for each variable; increment (Inc) of increase specified. Adjustment was performed for gestation at recruitment, estimated fetal weight centile and amniotic fluid index. Variables in bold text indicate independently predictive variables (aOR p<0.05). Birth weight and estimated fetal weight centiles calculated by Bulk centile calculator v6.7 (UK) (Gestation Network, Birmingham, UK). UAD = Umbilical artery Doppler. MCA = Middle cerebral artery Doppler. PI = Pulsatility Index. RI = Resistance Index.

**Fig 4:**
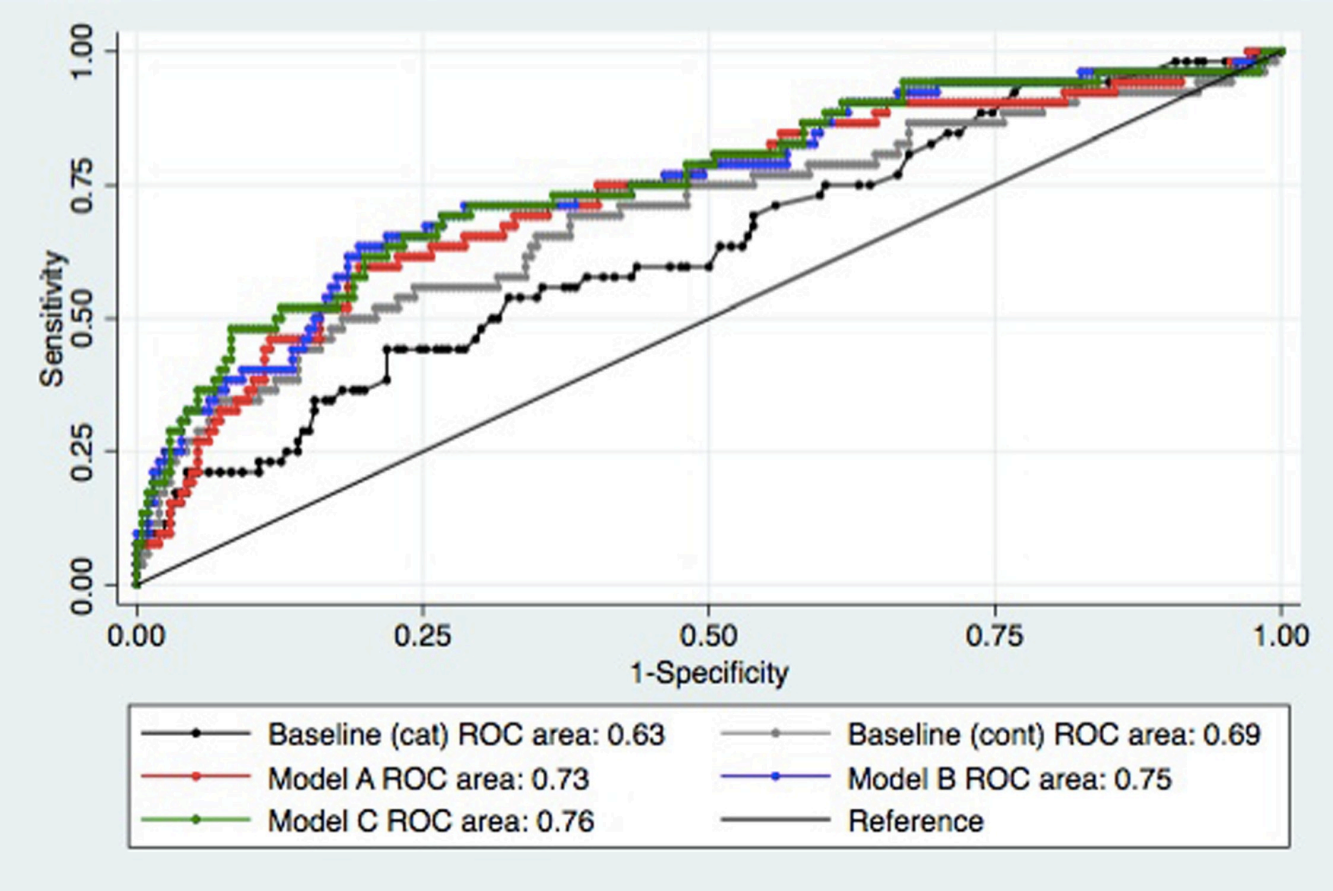
Receiver operator characteristic curve comparison. Demonstrating model performance in predicting adverse pregnancy outcome (APO) for the baseline and proposed models (see Table 3 for model components) in N = 258* pregnancies, of whom 52 (20.2%) experienced APO. The proposed models were superior to the baseline models (p<0.05). AUC = area under receiver operating characteristic curve. * maternal blood sample unavailable in 36 cases, amniotic fluid index measurement unavailable in 2 cases.

Recognising that in practice, current care largely considers EFW centile and amniotic fluid index in categorical terms (abnormal if <10^th^ centile and <5^th^ centile respectively) the performance of this “categorical” baseline care was also assessed. This performance of this model was significantly worse than the “continuous” baseline model (categorical ROC area 0.61 (95%CI 0.53–0.69) vs. continuous ROC area 0.69 (0.60–0.78), p = 0.031). We therefore used the EFW centile and amniotic fluid index as continuous variables in our baseline care model. Multiple logistic regression with backward elimination (whereby the variable(s) contributing least to any given combination of variables was removed at each iteration until the new model lost significance compared with the baseline model or the previous proposed model) identified three potentially useful novel predictive models (Table 3). Fig 4 shows the ROC curves for baseline “continuous” and proposed models.

**Table 3:** Components and comparison of proposed predictive models.

Model	Logit(pAPO) =	N	AUC	95%CI
**Baseline** (categorical)	6.88–1.52*log(Gest) + 1.62*(EFW centile <10)– 0.57*(Amniotic fluid index centile <5)	296	0.61	0.53–0.69
**Baseline** (continuous)	20.82–3.26*log(Gest)– 0.31*√(EFW centile)– 0.62*√(Amniotic fluid index)	294	0.69	0.60–0.78
**Model 1**	32.86–5.98*log(Gest)– 0.30*√(EFW centile)– 0.46*log(PlGF/sFlt-1)	258	0.73	0.65–0.81
**Model 2**	18.40–3.37*log(Gest)– 0.27*√(EFW centile)– 0.45*log(PlGF/sFlt-1) + 2.91*log(UAD-free PI)	258	0.75	0.67–0.83
**Model 3**	9.33–2.70*log(Gest)– 0.27*√(EFW centile)– 0.47*log(PlGF/sFlt-1) + 8.45*(UAD-free RI)	258	0.76	0.68–0.84

Compared with the baseline models (categorical or continuous), the proposed models display significantly higher Receiver Operator Curve (ROC) area (p<0.05). Key: pAPO = probability of adverse pregnancy outcome. Logit (pAPO) = ln(pAPO/1-pAPO). AUC = ROC area under curve. Gest = gestation (days) at recruitment. EFW = estimated fetal weight (centile calculated by Bulk centile calculator v6.7 (UK) (Gestation Network, Birmingham, UK). UAD-Free = umbilical artery free loop Doppler. PI = pulsatility index. RI = resistance index. PlGF = total placental growth factor concentration in maternal serum (pg/ml). sflt-1 = total soluble fms-like tyrosine kinase concentration in maternal serum (pg/ml).

The ROC area of models combining Doppler measures with PlGF/sFlt-1 ratio & baseline care (models B and C) were not significantly better than Model A (PlGF/sFlt-1 ratio n& baseline care; p = 0.24–0.28). A potential model combining baseline care with PlGF/sFlt-1 ratio and Middle Cerebral Artery Doppler/UAD-Free RI ratio did demonstrate statistical significance over baseline care (ROC area 0.76, p = 0.016) but was rejected due to significant risk of over-fitting (44 adverse pregnancy outcomes in N = 189 cases) and did not demonstrate superiority over Model A (p = 0.45). Test characteristics for proposed models are presented with their maximal sensitivity and negative predictive values (Table 4).

**Table 4:** Test performance characteristics of predictive models.

Model	Sensitivity (%)	Specificity (%)	PPV (%)	NPV (%)	LR+	LR-	Post-test probability (%)	
							Positive	Negative
Baseline Categorical* N = 296 APO = 61	27.9 (18.2–40.2)	85.5 (80.5–89.5)	33.3 (22.0–47.0)	82.0 (76.8–86.3)	1.9 (1.2–3.2)	0.84 (0.72–0.99)	74.2 (44.6–98.3)	18.9 (17.7–20.4)
Baseline Continuous* N = 294 APO = 60	21.7 (13.1–33.6)	96.6 (93.4–98.3)	61.9 (40.9–79.3)	82.8 (77.9–86.8)	6.3 (2.8–14.6)	0.81 (0.71–0.93)	68.9 (49.6–83.7)	16.7 (14.6–19.2)
**Model A**^#^ N = 258 APO = 52	26.4 (16.4–39.6)	94.2 (90.1–96.7)	53.9 (35.5–71.2)	83.9 (78.0–87.6)	4.6 (2.2–9.3)	0.78 (0.66–0.92)	61.5 (43.6–76.6)	16.1 (13.6–19.0)
**Model B**^#^ N = 258 APO = 52	32.1 (21.1–45.5)	95.7 (91.9–97.7)	65.4 (46.2–80.6)	84.6 (79.4–88.7)	7.4 (3.5–15.6)	0.71 (0.59–0.86)	72.2 (55.2–84.6)	14.6 (12.2–17.7)
**Model C**^#^ N = 258 APO = 52	35.8 (24.4–49.3)	94.7 (90.7–97.0)	63.3 (45.5–78.1)	85.2 (80.0–89.2)	6.7 (3.4–13.3)	0.68 (0.55–0.83)	70.2 (54.4–82.4)	14.0 (11.3–17.2)

Test characteristics are presented at optimal test characteristics and are displayed with 95% confidence intervals for each predictive model. See Table 3 for model composition. Key: PPV/NPV = positive and negative predictive values. LR+/LR- = positive and negative likelihood ratios. Positive post-test probability = probability of adverse pregnancy outcome (APO) following a positive test by each predictive model. Negative post-test probability = probability of APO following a negative test by each predictive model.

* Full amniotic fluid index missing for N = 2 individuals.

^#^ Maternal serum donation refused by N = 36 individuals

Compared with the baseline models, each proposed model demonstrated superior test performance statistics, however the 95% confidence intervals overlapped significantly. Consequently, there was no significant improvement in the number needed to screen to detect an additional case of adverse outcome (compared with baseline detection) with any proposed model (p>0.05). Furthermore, despite positive likelihood ratio>4 for each model with post-positive test probability of adverse pregnancy outcome of ≥61.5%, no model achieved negative likelihood ratio<0.2, resulting in a significant residual post-negative test adverse pregnancy outcome probability. There was no significant alteration in the ROC areas generated by any model when limited to women who presented >37 weeks (30/152 pregnancies adverse pregnancy outcome ≥37 weeks; Table 5). Furthermore the odds ratios associated with EFW centile and PlGF/sFlt-1 were comparable >37 weeks, although UAD did not retain significance.

**Table 5:** Model performance by gestational age at presentation.

Model	AUC	Odds Ratios for adverse pregnancy outcome per unit change					Sens (%)	NPV (%)
		EFW (per 5 centiles)	Amniotic fluid index (per cm)	Log[PlGF/sFlt-1] (per log change)	UAD PI (per 0.1 units)	UAD RI (per 0.1 units)		
**All gestations**								
Baseline Categorical* N = 296	0.61	5.05* (2.00–12.75) **p = 0.001**	1.76* (0.07–42.09) p = 0.64				27.9 (17/61)	82.0 (229/279)
**Baseline** **Continuous** **N = 294**	0.69	0.89 (0.84–0.94) p<0.001	0.90 (0.82–0.99) p = 0.051				21.7 (13/60)	82.8 (226/273)
**Model A** **N = 260**	0.73	**0.89** **(0.84–0.94)** **p<0.001**		**0.63** **(0.47–0.84)** **p = 0.002**			26.9 (14/52)	83.9 (198/236)
**Model B** **N = 258**	0.75	**0.90** **(0.85–0.96)** **p<0.001**		**0.64** **(0.47–0.86)** **p = 0.003**	**1.37** **(1.12–1.68)** **p = 0.004**		23.1 (12/52)	83.5 (203/243)
**Model C** **N = 258**	0.76	**0.90** **(0.85–0.96)** **p<0.001**		**0.63** **(0.47–0.85)** **p = 0.003**		**2.40** **(1.41–4.10)** **p = 0.002**	25.0 (13/52)	84.0 (204/243)
**≥37 weeks’ gestation**								
Baseline Categorical* N = 152	0.57	**3.80** **(1.16–12.43)**	U/A				40.0 (12/30)	62.9 (88/140)
**Baseline** **Continuous** **N = 151**	0.70	**0.89** **(0.83–0.96)** **p = 0.002**	0.94 (0.82–1.08) p = 0.39				20.7 (6/29)	83.7 (118/141)
**Model A** **N = 130**	0.77	**0.90** **(0.83–0.97)** **p = 0.005**		**0.50** **(0.30–0.82)** **p = 0.006**			36.0 (9/25)	86.2 (100/116)
**Model B** **N = 130**	0.78	**0.91** **(0.83–0.99)** **p = 0.012**		**0.52** **(0.31–0.87)** **p = 0.012**	1.24 (0.90–1.73) p = 0.24		32.0 (8/25)	85.5 (100/117)
**Model C** **N = 130**	0.78	**0.90** **(0.83–0.98)** **p = 0.010**		**0.53** **(0.32–0.87)** **p = 0.013**		1.76 (0.76–4.07) p = 0.20	32.0 (8/25)	85.5 (100/117)

Demonstrating the relative predictive performance of elements of the established models (see Table 3 for model composition) for adverse pregnancy outcome within the whole cohort and after 37 weeks’ gestation. Odds ratios are presented per specified unit change, except for the Baseline Categorical model (*) where estimated fetal weight above or below the 10^th^ centile, and amniotic fluid index above or below the 5^th^ centile are treated as binary options. In the ≥37 week cohort only 2 individuals had AFI <5^th^ centile (1 adverse outcome) and therefore it was not possible to assess the odds of adverse outcome in this group. The contribution of PlGF/sFlt-1 remains relatively constant even at term gestations. Key: AUC = area under receiver operator curve. EFW = estimated fetal weight. PlGF/sFlt-1 = ratio of maternal serum placental growth factor and soluble fms-like tyrosine kinase concentrations. UAD = umbilical artery Doppler (free loop). PI = pulsatility index. RI = resistance index. Sens = sensitivity. NPV = negative predictive value. U/A = unable to assess.

## Discussion

Our findings provide limited support for the hypothesis that antenatal placental assessment has the potential to assist detection of RFM pregnancies at highest risk of adverse pregnancy outcome (of placental origin) compared to current care. However, use of a practical, although imprecise, definition of adverse outcome and the sensitivity/reliability of currently available tests of placental dysfunction do not justify immediate clinical application. In particular, PlGF/sFlt-1 ratio is shown as a promising biomarker of placental dysfunction and deserves further development and evaluation in this context.

A high rate of induction of labour is noted across the whole cohort (44%), likely reflecting an increased awareness of the high risk nature of this population in the base hospital (where previous RFM research has been performed [15, 17, 31, 32]). In other units a more selective elective delivery policy may have been employed [10], which may have altered the observed pregnancy outcomes between the two groups. The lack of statistically significant difference in caesarean section rates between the two groups may additionally reflect the effect of 24 hour on site obstetric consultant presence of the base hospital on rates of emergency caesarean deliveries [37]. This is supported by the significantly higher rate of emergency delivery for fetal distress in the adverse pregnancy outcome group reflecting a higher rate of assisted vaginal delivery in these pregnancies.

Two placental abruptions occurred, both in the adverse outcome group. One was associated with fulminant preeclampsia and resulted in stillbirth eight days after presentation with RFM at 31 weeks. The other resulted in emergency caesarean section and delivery of a severely compromised infant in the absence of hypertensive complications 46 days after presentation with RFM. Both cases demonstrated a unilateral high resistance uterine artery Doppler waveform at presentation with RFM and likely reflect maternal-origin impaired placental implantation rather than placental dysfunction *per se*.

The principal strength of this study is the multi-domain prospective assessment of *in utero* placental structure and function in the context of a common antenatal complaint, within a diverse population with high quality data acquisition. The facility and expertise to measure these aspects of placental structure and function exist in high income countries’ worldwide, making the model(s) widely implementable. Use of clinical parameters (such as EFW centile, amniotic fluid index and UAD impedance) as continuous variables results in more favourable test performance characteristics for predicting adverse pregnancy outcome than use of categorical variables (such as EFW centile<10, amniotic fluid index <5^th^ centile, UAD impedance >95^th^ centile). This fits with the knowledge that many term infants experiencing chronic placental insufficiency displayed apparently “normal” clinical features, such as UAD impedance <95^th^ centile [38–40], even in those resulting in stillbirth [41]. Risk calculators dealing with multiple continuous variables may be of higher clinical utility than classical “cut offs”.

Previous studies have reported enhanced prediction of adverse pregnancy outcome amongst high-risk pregnancies using limited structural, vascular and endocrine placental assessment in the first [8], and second [7, 42], trimesters. Here, we demonstrate that multifaceted placental assessment later in pregnancy (when delivery is feasible) is possible and potentially useful. Although the performance of the prediction models was modest in this study, the association between an altered angiogenic marker balance was consistent across all the prediction models and, importantly, retained in women presenting >37 weeks. We believe these findings represent a key step in narrowing the scope for future research in this area, including better understanding of the relationship between placental dysfunction and the PlGF/sFlt-1 ratio, particularly in the late third trimester.

Low maternal PlGF [43, 44], and high maternal sFlt-1 concentrations [45–49] in pregnancies resulting in adverse pregnancy outcome have been previously described. Furthermore, we previously demonstrated increased villus release of sFlt-1 in placentas from RFM pregnancies with adverse pregnancy outcome [15], while Benton *et al*. [50] have shown high-grade histological placental insufficiency in pregnancies with PlGF <5^th^ centile. Additionally Ukah *et al*. highlighted that the principal role of PlGF-based tests within the hypertensive diseases of pregnancy population is in the prediction of adverse fetal (placental) outcomes [51] and Griffin *et al*. [36] also demonstrated increased prediction of small for gestational age birth when PlGF measurement was combined with EFW centile.

We were unable to replicate the potential predictive value of hPL, hCG or diastolic blood pressure for adverse pregnancy outcome following RFM that we previously reported[31]. This may relate to exclusion of premature birth from our definition of adverse pregnancy outcome in this study. Furthermore, our failure to corroborate, in the third trimester, the predictive value of uterine artery Doppler in RFM pregnancies previously shown in the first and second trimester [7, 8] may reflect late normalisation of uterine artery Doppler impedance [52–54].

A number of limitations are recognised, particularly use of a composite adverse pregnancy outcome definition [55], elements of which may have been censored by obstetric intervention. The prognostic significance of birth weight centile <10 is uncertain [56–58] and may have resulted in incorrect classification of constitutionally small fetuses and those with declining growth trajectory. It is likely that predictive accuracy of placental assessment would be significantly improved with a more robust/precise definition of adverse pregnancy outcome.

We acknowledge the potential for bias to have been introduced to this study in two key stages. Firstly, there is no searchable record kept by the hospital of all presentations with RFM in the study period (as there is no clinical code for RFM). Thus the number of potential participants ineligible due to immediate fetal compromise is unknown, as is the number of potentially eligible participants who were not referred to the research team. Secondly, bias may have been introduced by the researcher conducting the ultrasound assessments not being blinded to the clinical history or conventional ultrasound results (EFW, UAD impedance) at the time of the other sonographic measurements being taken. However, this individual had no influence on the clinical care delivered to the participant following the research assessment and assessment of PlGF/sFlt1 ratio was performed blinded to clinical and sonographic details.

Furthermore, suboptimal intra-observer reliability [59] (e.g. placental volume [18]) and missing data (e.g. Middle Cerebral Artery impedance) may have resulted in premature rejection of potentially useful measures/models (for example rejection of the model that included cerebroplacental ratio). Improvement of such techniques, including standardised protocols and operator experience at obtaining such measurements at advanced gestation (such as has been successfully achieved in the case of Middle Cerebral Artery [60]) may improve clinical utility. The cerebroplacental ratio has shown promise for the prediction of fetal compromise in previous studies [61] which have not reported the rate of missing data [60]. Explanations for the high rate of missing data for this variable in the current study may include more stringent rejection of suboptimal insonation angles, or limited scan duration in our study compared with other authors’ research protocols. The findings of the RATIO37 study are awaited [62].

## Conclusion

RFM is a commonly encountered problem in maternity services. Current care fails to prospectively identify many pregnancies subsequently ending in adverse pregnancy outcome after RFM. This study identified two clinical measures relating to placental health (UAD impedance and PlGF/sFlt1 ratio in maternal serum) that have the potential to incrementally improve prediction of adverse pregnancy outcome after RFM. However, these tests require further development and evaluation of their link to placental dysfunction and fetal wellbeing. The full diagnostic potential of these tests, particularly of the PlGF/sFlt1 ratio, needs to be prospectively assessed in future studies. Given the significant declining fetal weight centile for all RFM pregnancies (regardless of outcome category), the clinical benefit, and health economic impact, of interval scanning of pregnancies continuing after presentation with RFM should also be considered.

## Supporting information

S1 Table STARD checklist. The study was conducted in accordance to STARD guidelines.
(DOCX)

S2 Table Variable reduction. The distributions of variables between groups were compared by univariate analysis. Non-parametric data (M) are presented as median (IQR) and compared by Mann-Whitney U Test, parametric data (T) as mean ± standard deviation and compared by Students’ T Test and categorical data (C) as number (%) and compared by Chi squared test with Yates’ correction as required. Bold text denotes variables meeting a priori variable reduction criteria of p<0.10. Key: RFM = reduced fetal movements. CTG = cardiotocograph. Bpm = beats per minute. EFW = estimated fetal weight (centile calculated by bulk centile calculator v6.7 (UK) (Gestation Network, Birmingham, UK)). CoV = coefficient of variance. UAD = umbilical artery Doppler. MCA = middle cerebral artery Doppler. PI = pulsatility index. RI = resistance index. hCG = human chorionic gonadotrophin. hPL = human placental lactogen. PlGF = placental growth factor. sFlt-1 = soluble fms-like Tyrosine Kinase-1. ^¥^ = Hormone concentration / sonographic placental volume. ^♯^ = Free-PlGF calculated as PlGF x (PlGF/sFlt-1).
(DOCX)
