# Supplementary material for: Antenatal placental assessment in the prediction of adverse pregnancy outcome after reduced fetal movement
Source: PLoS One. 2018 Nov 5;13(11):e0206533. doi: 10.1371/journal.pone.0206533 (PMC6218043; doi:10.1371/journal.pone.0206533)
Supplement: S2 Table — The distributions of variables between groups were compared by univariate analysis. Non-parametric data (M) are presented as median (IQR) and compared by Mann-Whitney U Test, parametric data (T) as mean ± standard deviation and compared by Students’ T Test and categorical data (C) as number (%) and compared by Chi squared test with Yates’ correction as required. Bold text denotes variables meeting a priori variable reduction criteria of p<0.10. Key: RFM = reduced fetal movements. CTG = cardiotocograph. Bpm = beats per minute. EFW = estimated fetal weight (centile calculated by bulk centile calculator v6.7 (UK) (Gestation Network, Birmingham, UK)). CoV = coefficient of variance. UAD = umbilical artery Doppler. MCA = middle cerebral artery Doppler. PI = pulsatility index. RI = resistance index. hCG = human chorionic gonadotrophin. hPL = human placental lactogen. PlGF = placental growth factor. sFlt-1 = soluble fms-like Tyrosine Kinase-1. ¥ = Hormone concentration / sonographic placental volume. ♯ = Free-PlGF calculated as PlGF x (PlGF/sFlt-1). (DOCX) [file pone.0206533.s002.docx]

S2 Table: Variable reduction.

|  | **Pregnancy outcome** | **Normal** | **Adverse** | **p** |
| --- | --- | --- | --- | --- |
|  | **Baseline model** | | | |
| ^M^ | Gestation (days) | 260 (235 – 275) | 258 (233 – 271) | 0.30 |
| **^M^** | **EFW centile** | **66 (44 – 86)** | **41 (18 – 65)** | **<0.0001** |
| ^C^ | **EFW centile <10** | **9/240 (4%)** | **12/56 (21%)** | **<0.0001** |
| **^M^** | **Amniotic Fluid Index cm^1^** | **11 (9 – 15)** | **11 (8 – 13)** | **0.088** |
| ^C^ | Amniotic fluid index <5^th^ centile | 3/234 (1%) | 1/61 (2%) | 0.83 |
|  | **Maternal characteristics** | | | |
| ^T^ | Age (years) | 29 ± 5 | 29 ± 6 | 0.60 |
| **^T^** | **Height (cm)** | **164 ± 7** | **163 ± 6** | **0.086** |
| ^M^ | Weight (kg) | 69 (60 – 81) | 70 (58-83) | 0.99 |
| ^M^ | BMI (kg/m^2^) | 25 (23 – 29) | 26 (23 – 30) | 0.20 |
| ^C^ | Smoker (current) | 30/235 (13%) | 8/61 (13%) | 0.94 |
| ^C^ | Alcohol intake (current) | 3/235 (1%) | 1/61 (2%) | 0.83 |
| ^C^ | Illegal drug use (current) | 0/235 (0%) | 0/61 (0%) | 1.00 |
| ^C^ | White European | 152/235 (65%) | 41/61 (67%) | 0.71 |
| ^C^ | Black | 31/235 (13%) | 6/61 (10%) | 0.48 |
| ^C^ | Asian | 39/235 (17%) | 11/61 (18%) | 0.79 |
| ^C^ | Other ethnicity | 13/235 (6%) | 3/61 (5%) | 0.85 |
| ^M^ | Parity (number) | 0 (0 – 1) | 0 (0 – 1) | 0.69 |
| **^M^** | **Miscarriages (number)** | **0 (0 – 1)** | **0 (0 – 1)** | **0.089** |
| ^M^ | Number of previous Caesarean Sections | 0 (0 – 0) | 0 (0 – 0) | 0.13 |
| **^C^** | **Previous birth weight<10^th^ centile** | **19/235 (8%)** | **10/61 (18%)** | **0.052** |
| ^C^ | Previous stillbirth | 2/235 (1%) | 1/61 (2%) | 0.58 |
| ^M^ | Booking Gestation (days) | 73 (64 – 84) | 74 (67 – 86) | 0.51 |
| **^C^** | **Significant Past Medical History** | **16/235 (7%)** | **9/61 (15%)** | **0.047** |
| ^C^ | Mental Health Disorder | 15/235 (6%) | 1/61 (2%) | 0.14 |
|  | **Features of RFM episode** | | | |
| ^M^ | Gestation (days) | 260 (235 – 275) | 258 (221 – 271) | 0.21 |
| ^C^ | Term gestation | 123/235 (52%) | 30/61 (49%) | 0.66 |
| ^M^ | Episode number | 1 (1 – 1) | 1 (1-1) | 0.72 |
| **^M^** | **Duration (hours)** | **36 (18 – 72)** | **48 (24 – 72)** | **0.073** |
| ^M^ | Absent (hours) | 0 (0 – 2) | 0 (0 – 3) | 0.56 |
| ^M^ | Systolic blood pressure (mm/Hg) | 111 (103 – 120) | 110 (102 – 122) | 0.95 |
| ^M^ | Diastolic blood pressure (mm/Hg) | 69 (60 -75) | 70 (62 – 80) | 0.1 |
| ^C^ | Proteinuria | 19/175 (11%) | 6/49 (12%) | 0.79 |
| ^T^ | CTG: Baseline rate (bpm)^2^ | 137 ± 8 | 136 ± 8 | 0.44 |
| ^C^ | CTG: Variability <5bpm^2^ | 0/204 (0%) | 0/53 (0%) | 1.00 |
| ^C^ | CTG: Acceleration^2^ | 192/204 (94%) | 48/53 (91%) | 0.35 |
| ^M^ | CTG: Movements /min | 0.28 (0.12 – 0.69) | 0.31 (0.11 – 0.57) | 0.47 |
| ^C^ | RFM resolved | 95/216 (44%) | 21/56 (38%) | 0.38 |
|  | **Placental assessment** | | | |
| ^C^ | Anterior placenta | 82/235 (35%) | 26/61 (43%) | 0.26 |
| ^C^ | Lateral placenta | 66/235 (28%) | 16/61 (26%) | 0.77 |
| ^C^ | Posterior placenta | 49/235 (21%) | 10/61 (16%) | 0.44 |
| ^C^ | Fundal placenta | 38/235 (16%) | 9/61 (15%) | 0.79 |
| **^T^** | **Length (cm)^3^** | **18.7 ± 2.7** | **17.9 ± 3.0** | **0.035** |
| **^T^** | **Width (cm)^3^** | **15.8 ± 2.7** | **15.1 ± 3.1** | **0.080** |
| ^M^ | Width:Length | 0.86 (0.78 – 0.93) | 0.86 (0.80 – 0.94) | 0.73 |
| ^M^ | Depth (cm)^3^ | 4.3 (3.7 – 5.0) | 4.1 (3.6 – 5.0) | 0.78 |
| ^M^ | Length:Depth | 4.3 (3.6 – 5.1) | 4.2 (3.5 – 5.1) | 0.36 |
| ^M^ | Width:Depth | 3.7 (3.0 – 4.4) | 3.4 (3.0 – 4.3) | 0.18 |
| ^M^ | Volume (cm^3^)^3^ | 477 (364 – 602) | 445 (321 – 588) | 0.21 |
| ^C^ | Eccentric cord (qualitative) | 53/193 (26%) | 9/48 (23%) | 0.22 |
| ^T^ | Cord distance ratio | 0.32 ± 0.01 | 0.33 ± 0.01 | 0.72 |
|  | **Placental vascular assessment** | | | |
| **^M^** | **UAD-Abdomen PI** | **0.93 (0.80 – 1.13)** | **1.03 (0.92 – 1.28)** | **0.0054** |
| **^T^** | **UAD-Abdomen RI** | **0.62 ± 0.09** | **0.68 ± 0.10** | **0.0003** |
| **^M^** | **UAD-Free PI** | **0.88 (0.76 – 1.04)** | **0.99 (0.84 – 1.13)** | **0.0010** |
| **^T^** | **UAD-Free RI** | **0.59 ± 0.08** | **0.63 ± 0.09** | **0.004** |
| ^M^ | UAD-Placenta PI | 0.80 (0.68 – 0.93) | 0.83 (0.72 – 0.94) | 0.36 |
| ^T^ | UAD-Placenta RI | 0.55 ± 0.08 | 0.55 ± 0.09 | 0.45 |
| ^M^ | Chorionic plate artery PI | 0.78 (0.69 – 0.89) | 0.80 (0.73 – 0.92) | 0.13 |
| **^T^** | **Chorionic plate artery RI** | **0.53 ± 0.06** | **0.55 ± 0.06** | **0.044** |
| **^M^** | **Intraplacental artery PI** | **0.71 (0.62 – 0.79)** | **0.74 (0.70 – 0.82)** | **0.017** |
| **^T^** | **Intraplacental artery RI** | **0.50 ± 0.06** | **0.52 ± 0.06** | **0.043** |
| ^M^ | UAD-Free:UAD-Abdomen PI | 1.10 (0.95 – 1.20) | 1.04 (0.89 – 1.21) | 0.40 |
| ^M^ | UAD-Free:UAD-Abdomen RI | 1.05 (0.98 – 1.12) | 1.03 (0.97 – 1.14) | 0.45 |
| ^M^ | UAD-Placenta:UAD-Abdomen PI | 1.17 (1.00 – 1.33) | 1.24 (0.99 -1.43) | 0.28 |
| ^M^ | UAD-Placenta:UAD-Abdomen RI | 1.13 (1.02 – 1.22) | 1.19 (1.00 – 1.13) | 0.17 |
| **^M^** | **Chorionic plate artery PI : UAD-Abdomen PI** | **1.16 (1.02 – 1.35)** | **1.30 (1.07 – 1.40)** | **0.086** |
| ^M^ | Chorionic plate artery PI : UAD-Abdomen RI | 1.15 (1.05 – 1.25) | 1.19 (1.09 – 1.28) | 0.14 |
| ^M^ | Intraplacental artery PI : UAD-Abdomen PI | 1.31 (1.12 – 1.55) | 1.29 (1.20 – 1.69) | 0.31 |
| ^T^ | Intraplacental artery PI : UAD-Abdomen RI | 1.23 ± 0.19 | 1.28 ± 0.17 | 0.20 |
| **^M^** | **UAD-Placenta:UAD-Free PI** | **1.09 (0.97 – 1.29)** | **1.19 (1.01 – 1.37)** | **0.074** |
| **^M^** | **UAD-Placenta:UAD-Free RI** | **1.07 (0.98 – 1.19)** | **1.10 (1.02 - 1.23)** | **0.091** |
| **^M^** | **Chorionic plate artery PI : UAD-Free PI** | **1.11 (0.98 – 1.25)** | **1.21 (1.05 – 1.32)** | **0.018** |
| **^M^** | **Chorionic plate artery PI : UAD-Free RI** | **1.11 (1.02 - 1.18)** | **1.14 (1.06 – 1.23)** | **0.064** |
| ^M^ | Intraplacental artery PI : UAD-Free PI | 1.22 (1.08 – 1.40) | 1.31 (1.05 – 1.50) | 0.18 |
| ^M^ | Intraplacental artery RI : UAD-Free RI | 1.15 (1.06 – 1.28) | 1.20 (1.07 – 1.30) | 0.28 |
| ^M^ | Chorionic plate artery PI : UAD-Placenta PI | 1.01 (0.89 – 1.15) | 1.03 (0.91 – 1.18) | 0.78 |
| ^M^ | Chorionic plate artery PI: UAD-Placenta RI | 1.02 (0.94 – 1.10) | 1.02 (0.91 – 1.11) | 0.78 |
| ^M^ | Intraplacental artery PI : UAD-Placenta PI | 1.11 (0.99 – 1.29) | 1.09 (0.94 – 1.28) | 0.62 |
| ^M^ | Intraplacental artery : UAD-Placenta RI | 1.08 (0.99 – 1.19) | 1.07 (0.99 – 1.17) | 0.58 |
| ^M^ | Intraplacental artery PI : Chorionic plate artery PI | 1.11 (0.99 – 1.24) | 1.09 (1.01 – 1.20) | 0.80 |
| ^M^ | Intraplacental artery RI : Chorionic plate artery RI | 1.07 (0.98 – 1.14) | 1.07 (1.00 – 1.14) | 0.86 |
| ^M^ | Uterine artery Doppler PI | 0.78 (0.63 – 1.02) | 0.87 (0.68 – 1.11) | 0.17 |
| ^M^ | Uterine artery Doppler RI | 0.51 (0.44 – 0.58) | 0.54 (0.46 – 0.62) | 0.21 |
|  | **Placental endocrine assessment** | | | |
| ^M^ | hCG (mIU/ml) | 380 (284 – 507) | 418 (294 – 562) | 0.31 |
| ^M^ | Normalised hCG (mIU/ml/cm)^3¥^ | 0.80 (0.54 – 1.24) | 1.05 (0.54 – 1.58) | 0.14 |
| **^M^** | **hPL (mg/ml)** | **738 (562 – 922)** | **661 (539 – 794)** | **0.044** |
| ^M^ | Normalised hPL (mg/ml/cm^3^) **^¥^** | 1.55 (1.15 – 2.08) | 1.42 (1.16 – 1.89) | 0.98 |
| ^M^ | Progesterone (ng/ml) | 812 (529 – 1121) | 726 (537 – 1025) | 0.69 |
| ^M^ | Normalised Progesterone (ng/ml/cm^3^) **^¥^** | 1.70 (1.12 – 2.52) | 1.79 (1.14 – 2.50) | 0.54 |
| **^M^** | **PlGF (pg/ml)** | **343 (211 – 607)** | **277 (156 – 410)** | **0.025** |
| ^M^ | Normalised PlGF (pg/ml/cm^3^) **^¥^** | 0.73 (0.46 – 1.29) | 0.53 (0.33 – 1.48) | 0.30 |
| ^M^ | sFlt-1 (pg/ml) | 1785 (1030 – 3562) | 2562 (1008 – 4209) | 0.27 |
| ^M^ | Normalised sFlt-1 (pg/ml/cm^3^) **^¥^** | 3.79 (1.93 – 8.17) | 5.13 (2.59 – 9.86 | 0.14 |
| **^M^** | **PlGF:sFlt-1** | **0.20 (0.06 – 0.61)** | **0.10 (0.04 – 0.38)** | **0.052** |
| ^M^ | Normalised PlGF:sFlt-1 (1/cm^3^) **^¥^** | 0.41 (0.12 – 1.10) | 0.20 (0.10 – 0.82) | 0.17 |
| **^M^** | **Free-PlGF^♯^ (pg/ml)** | **74 (13 – 322)** | **21 (8 – 155)** | **0.038** |
| ^M^ | Normalised Free-PlGF (pg/ml/cm^3^) **^¥^** | 0.13 (0.022 – 0.56) | 0.046 (0.017 – 0.28) | 0.13 |
|  | **Brachiocephalic blood diversion** | | | |
| ^M^ | MCA PI | 1.60 (1.25 – 1.96) | 1.67 (1.38 – 1.83) | 0.67 |
| ^T^ | MCA RI | 0.77 ± 0.09 | 0.77 ± 0.08 | 0.85 |
| ^T^ | MCA Peak Systolic Velocity (cm/s) | 51 ± 14 | 52 ± 14 | 0.75 |
| **^M^** | **MCA:UAD-Abdomen PI** | **1.68 (1.35 – 1.98)** | **1.49 (1.23 – 1.85)** | **0.073** |
| **^M^** | **MCA:UAD-Abdomen RI** | **1.25 (1.15 – 1.37)** | **1.14 (1.05 – 1.28)** | **0.0051** |
| **^M^** | **MCA:UAD-Free PI** | **1.77 (1.48 – 2.07)** | **1.61 (1.21 – 2.00)** | **0.070** |
| **^M^** | **MCA:UAD-Free RI** | **1.31 (1.19 – 1.44)** | **1.23 (1.11 – 1.31)** | **0.0033** |
| ^M^ | MCA:UAD-Placenta PI | 1.95 (1.69 – 2.33) | 2.01 (1.52 – 2.31) | 0.71 |
| ^M^ | MCA:UAD-Placenta RI | 1.40 (1.30 – 1.52) | 1.41 (1.23 – 1.56) | 0.83 |
| ^M^ | MCA PI : Chorionic plate artery PI | 1.97 (1.72 – 2.36) | 1.90 (1.65 – 2.49) | 0.60 |
| ^T^ | MCA RI : Chorionic plate artery RI | 1.45 ± 0.18 | 1.41 ± 0.19 | 0.24 |
| ^M^ | MCA RI : Intraplacental artery PI | 2.19 (1.81 – 2.57) | 2.13 (1.77 – 2.46) | 0.29 |
| **^T^** | **MCA RI : Intraplacental artery RI** | **1.53 ± 0.20** | **1.47 ± 0.20** | **0.083** |
